# Dental Morbidity Among School-Going Adolescents in a Fluoride-Endemic Belt of Rural Andhra Pradesh

**DOI:** 10.7759/cureus.113028

**Published:** 2026-07-20

**Authors:** Anu Jose, Deepthi Kattula, Joanna Priyadarshini, Jomi Porinchu, Navadeep D Medabalimi

**Affiliations:** 1 Dental Unit, Christian Medical College Vellore, Chittoor Campus, Vellore, IND; 2 Dental Services, Betsi Cadwaladr University Health Board, Wales, GBR; 3 Department of Anatomy and Embryology, Ruhr University Bochum, Bochum, DEU

**Keywords:** adolescents, andhra pradesh, chittoor, dental caries, fluoride endemic, fluorosis, health utilization, oral hygiene

## Abstract

Background: Oral health problems are one of the major public health concerns that predominantly affect children, thereby affecting their general health, self-esteem, and quality of life. This study aimed to estimate the burden of dental caries, gingivitis, malocclusion, and fluorosis and understand healthcare utilization patterns and oral hygiene practices among school-going adolescents in a fluoride-endemic belt in the rural areas of Chittoor district, Andhra Pradesh, India.

Methods: Cross-sectional survey was conducted among 10-15 years aged school children in Gudipala Mandal of Chittoor district. Data on dental morbidity, oral health practices, and health utilization were collected. Categorical variables were presented as frequencies, and factors associated with dental caries were assessed using the chi-square test and adjusted for confounders using multiple logistic regression.

Results: Among the 808 study participants, the proportion of dental caries was 36%, gingivitis 12%, fluorosis 29%, and malocclusion 6.3%. Good oral hygiene was observed in 68% of the study participants. Health-seeking behavior was reported by only 2.2% of the participants. Older children aged 13-15years [OR: 0.72 (95% CI: 0.53-0.98)], good oral hygiene [OR: 0.57 (95% CI: 0.42-0.78)], and mild fluorosis [OR: 0.72 (95% CI: 0.52-1)] were independently protective against dental caries.

Conclusion: The overall burden of dental caries and gingivitis was less, but health utilization was poor, indicating the need to bring awareness about healthcare facilities and the importance of seeking treatment in the local community. Health systems should support capacity building of primary care facilities to make dental treatment accessible, available, and affordable and aid in establishing proper referral pathways for seeking secondary care if necessary.

## Introduction

Globally, oral health problems are still persistent among underprivileged communities of both developed and developing countries despite the vast improvements in healthcare delivery system. The World Health Organization (WHO) identifies dental caries as a major public health problem and reports that the prevalence of dental caries among school-aged children is 60-90% [1]. Children are the future of any country as they constitute an average of 25-28% of the total world population [2]; hence, their well-being and health impact the future of the nation. It has been noted that children with poor oral health from poorer socioeconomic background are 12 times more likely to have higher absenteeism than their healthy counterparts [3]. Children are vulnerable due to their lack of knowledge on oral hygiene, dietary habits, and motivation. Untreated dental problems affect the quality of life, contributing to pain, discomfort, and general well-being. Most oral health conditions, such as dental caries and gingival and periodontal diseases leading to tooth loss, are largely preventable and can be treated in their early stages if detected early.

Although dental caries is widely prevalent, its distribution and severity vary across countries and regions within a country because of the interplay of several factors such as socioeconomic status, oral hygiene, level of parental education, dietary patterns, access to health care, and exposure to different levels of fluoride [4]. Fluoride is a natural mineral but acts like a double-edged sword, a boon for the prevention of dental caries by strengthening enamel, enhancing remineralization, and reducing the risk of acid-induced demineralization, and it is a bane when present in excessive amounts, causing dental and skeletal fluorosis [5]. The desired level is often considered around 1.0 mg/L for dental health benefits, although optimal ranges (0.5-1.0 mg/L) vary with climate [6]. The WHO sets an upper limit for fluoride in drinking water at 1.5 mg/L [6]. A report from UNICEF in India documented the endemicity of fluorosis in Andhra Pradesh, Telangana, and Rajasthan, with the highest fluoride content of 8.6 ppm, 18.5 ppm, and 16.9 ppm, respectively [7,8]. While a few studies have been conducted on dental caries and dental fluorosis in school children from certain fluoride-endemic districts of Andhra Pradesh [9,10], there is limited literature on oral health status from the rural region of Chittoor district.

Therefore, this study was conducted to estimate the burden of dental caries, gingivitis, and malocclusion and to understand the health utilization pattern and oral hygiene practices among school-going adolescents in a fluoride-endemic belt in the rural areas of Chittoor district, Andhra Pradesh, India. This study will enable us to understand the local community with respect to dental health and oral health practices and accordingly cater their needs efficiently and effectively.

## Materials and methods

This cross-sectional study was conducted among school-going children aged 10-15 years in Gudipala Mandal of Chittoor district as a part of a secondary care hospital outreach program that provides health education, general health, and dental evaluations. All government and government-aided schools in the vicinity of the secondary care hospital were identified, and permission was obtained from the District Education Officer and the respective school principals to conduct the health checkup. The school authorities informed the parents, and informed consent was obtained from the parents and assent from the adolescents. Children with special needs and those who did not give the assent were excluded from the study. A total sample size of 800 was estimated after considering the prevalence of dental caries to be 47% [11], a 5% type I error, an absolute precision of 5%, and a design effect of 2. Schools were enrolled in the study till sample size was attained.

The causes of tooth decay and proper brushing techniques were taught, followed by an oral examination using a mouth mirror and dental probe in a well-lit classroom. Documentation of dental caries, gingivitis, malocclusion, oral hygiene practices, and information on previous dental consultations, and availed treatments were recorded in a proforma. Data collection was performed by a dedicated dentist over a period of one year.

Any tooth that, on visual examination, showed discoloration with a broken surface was considered decayed or carious [12]. Gingivitis was diagnosed based on bleeding from the gums or redness (erythema) with or without swelling [13]. Any soft material on the tooth surface was considered debris, and any hard deposit was considered calculus [13]. Deciduous teeth present in the oral cavity beyond the time of exfoliation were marked as retained teeth [14]. Any deviation from ideal occlusion was considered malocclusion [15]. The presence or absence of habits was also noted. A tooth was considered fractured if it had a history of trauma with broken margins/edges. Children requiring any dental intervention were encouraged to visit the outpatient facility for appropriate guidance and management of their symptoms. The Institutional Review Board Ethics and Research Committee approved the study (IRB Min No. 2506174).

Data were entered into an Excel spreadsheet (Microsoft Corp., Redmond, WA, USA) and analyzed using Stata software (StataCorp LLC, College Station, TX, USA). Descriptive statistics were presented as frequencies for categorical variables. Stratified analysis was done to assess the distribution of dental morbidities between gender and age groups. Factors associated with dental caries were assessed using the chi-square test and adjusted for confounders using multiple logistic regression.

## Results

A total of 808 children were enrolled in the study, comprising 512 (63.4%) girls, with 511 (63.2%) children belonging to the age group of 13-15 years. Dental caries (either in deciduous or permanent dentition) was present in 292 (36%) children, with predominance in permanent dentition (226, 28%). Comparatively, more of pit and fissure caries was identified in 248 (31%) children, whereas smooth surface caries was present in only 51 (6.3%). A total of 235 (29.1%) children showed signs of the mild form of non-pitted fluorosis. Among the study children, 98 (12%) had gingivitis, 51 (6.3%) of them had malocclusion. Good oral hygiene was prevalent among 552 (68%) children. Only 18 (2.2%) children had a history of previous dental consultation, while only 13 (1.6%) had restorations. The descriptions of dental morbidities, oral hygiene practices, and health-seeking behavior among the study children are presented in Tables 1, 2.

**Table 1:** Dental morbidity in study population (n=808).

Variable	Number	Percentage
Presence of dental caries		
Yes	292	36
No	516	64
Caries in deciduous dentition		
Yes	80	10
No	728	90
Caries in permanent dentition		
Yes	226	28
No	582	72
Pit and fissure caries		
Present	248	31
Absent	560	69
Smooth surface caries		
Present	51	6.3
Absent	757	93.7
Fluorosis		
Present	235	29.1
Absent	573	70.9
Gingivitis		
Present	98	12
Absent	710	88
Molar relation		
Class I	781	96.7
Class II	20	2.5
Class III	7	0.8
Horizontal incisor relationship		
Normal	777	96.2
Increased	16	2
Cross bite	14	1.7
Scissor bite	1	0.1
Vertical Incisor relationship		
Normal	787	97.4
Deep bite	14	1.7
End on	7	0.9
Malocclusion		
Present	51	6.3
Absent	757	93.7

**Table 2:** Oral hygiene practices and health-seeking behavior in the study population.

Variable	Number	Percentage
Brushing technique		
Finger	63	7.8
Brush	736	91.1
Others	9	1.1
Debris		
Present	114	14.1
Absent	694	85.9
Calculus		
Present	161	20
Absent	647	80
Stains		
Present	37	4.6
Absent	768	95.4
Oral hygiene		
Good	552	68
Fair	239	30
Poor	17	2
History of extraction		
Yes	4	0.5
No	804	99.5
History of restoration		
Yes	13	1.6
No	795	98.4
History of dental consultation		
Yes	18	2.2
No	790	97.8
Missing tooth due to dental caries		
Yes	2	0.2
No	806	99.8

In the gender-specific stratified analysis, girls had a slightly higher proportion of dental caries, especially in the permanent dentition (p<0.0001), whereas boys had a more dental caries in the deciduous dentition (p=0.009). Boys had better oral hygiene than girls (p=0.03) (Table 3).

**Table 3:** Gender-specific stratified analysis.

Variable	Male n (%)	Female n (%)	Chi-square value	p value
Presence of dental caries				
Yes	95 (32.1)	197 (38.5)	3.31	0.06
No	201 (67.9)	315 (61.5)		
Caries in deciduous dentition				
Yes	40 (13.5)	40 (7.8)	6.83	0.009
No	256 (86.5)	472 (92.2)		
Caries in permanent dentition				
Yes	59 (19.9)	167 (32.6)	14.97	<0.0001
No	237 (80.1)	345 (67.4)		
Pit and fissure caries				
Present	80 (27)	168 (32.8)	2.95	0.08
Absent	216 (73)	344 (67.2)		
Smooth surface caries				
Present	19 (6.4)	32 (6.2)	0.009	0.92
Absent	277 (93.6)	480 (93.8)		
Fluorosis				
Present	89 (30)	146 (28.5)	0.21	0.64
Absent	207 (70)	366 (71.5)		
Gingivitis				
Present	35 (11.8)	63 (12.3)	0.04	0.84
Absent	261 (88.2)	449 (87.7)		
Malocclusion				
Present	20 (6.76)	31 (6.0)	0.15	0.69
Absent	276 (93.24)	481 (94.0)		
Oral hygiene				
Good	214 (72.3)	338 (66.0)	6.41	0.03
Fair	80 (27.0)	159 (31.0)		
Poor	2 (0.7)	15 (3)		

Age-specific stratified analysis (Table 4) showed a higher caries burden in the deciduous teeth among the younger age group (p<0.0001) and in the permanent teeth among the older age group (p=0.001). Smooth surface caries was more prevalent in the 10- to 12-year age group (p<0.0001).

**Table 4:** Age-specific stratified analysis.

Variable	10-12 Years n (%)	13-15 Years n (%)	Chi-square value	p value
Presence of dental caries				
Yes	119 (40.1)	173 (33.9)	3.14	0.07
No	178 (59.9)	338 (66.1)		
Caries in deciduous dentition				
Yes	67 (22.6)	13 (2.5)	84.34	<0.0001
No	230 (77.4)	498 (97.5)		
Caries in permanent dentition				
Yes	62 (20.9)	164 (32.1)	11.73	0.001
No	235 (79.1)	347 (67.9)		
Pit and fissure caries				
Present	93 (31.3)	155 (30.3)	0.08	0.77
Absent	204 (68.7)	356 (69.7)		
Smooth surface caries				
Present	32 (10.8)	19 (3.7)	15.81	<0.0001
Absent	265 (89.2)	492 (96.3)		
Fluorosis				
Present	85 (28.6)	150 (29.3)	0.04	0.82
Absent	212 (71.4)	361 (70.7)		
Gingivitis				
Present	30 (10.0)	68 (13.3)	1.81	0.17
Absent	267 (90.0)	443 (86.7)		
Malocclusion				
Present	24 (8.0)	27 (5.3)	2.48	0.11
Absent	273 (92.0)	484 (94.7)		
Oral hygiene				
Good	211 (71.04)	341 (66.73)	1.63	0.44
Fair	80 (26.94)	159 (31.12)		
Poor	6 (2.02)	11 (2.15)		

In univariate analysis, the older age group, good oral hygiene, and fluorosis offered protection against dental caries, as shown in Table 5.

**Table 5:** Univariate analysis of factors associated with dental caries.

Variable	Dental caries, Present, n (%)	Dental caries, Absent, n (%)	Chi-squarevalue	Unadjusted OR (95% CI)	p value
Gender					
Male	95 (32.5)	201 (39)	3.31	1.32 (0.97-1.78)	0.06
Female	197 (67.5)	315 (61)			
Age group					
10-12 Years	119 (40.8)	178 (34.5)	3.14	0.76 (0.56-1.02)	0.07
13-15 Years	173 (59.2)	338 (65.5)			
Type of dentition					
Mixed	105 (36)	136 (26.4)	8.21	0.63 (0.46-0.86)	0.004
Permanent	187 (64)	380 (73.6)			
Oral hygiene					
Poor/fair	116 (39.7)	140 (27.1)	13.66	0.56 (0.41-0.76)	<0.0001
Good	176 (60.3)	376 (72.9)			
Malocclusion					
Present	16 (5.5)	35 (6.8)	0.53	0.79 (0.43-1.45)	0.46
Absent	276 (94.5)	481 (93.2)			
Dental fluorosis					
Absent	221 (75.7)	352 (68.2)	5.04	0.68 (0.49-0.95)	0.02
Present	71 (24.3)	164 (31.8)			
Tooth brushing					
Neem stick/finger	23 (7.9)	49 (9.5)	0.6	1.22 (0.73-2.05)	0.43
Brush	269 (92.1)	467 (90.5)			

In multivariate analysis, factors such as older age (13-15 years) [OR: 0.72 (95% CI: 0.53-0.98)], good oral hygiene [OR: 0.57 (95% CI: 0.42-0.78)], and fluorosis [OR: 0.72 (95% CI: 0.52-1)] were independently protective against dental caries (Table 6).

**Table 6:** Multivariate analysis of factors associated with dental caries.

Variable	Adjusted OR for dental caries (95% CI)	p value
Gender		
Male	1	0.08
Female	1.31 (0.96-1.78)	
Age		
10-12 Years	1	0.03
13-15 Years	0.72 (0.53-0.98)	
Oral hygiene		
Poor/fair	1	0.001
Good	0.57 (0.42-0.78)	
Fluorosis		
Absent	1	0.05
Present	0.72 (0.52-1)	

## Discussion

Oral diseases pose as a major public health concern considering the magnitude of its prevalence and incidence. This study was undertaken to understand the patterns of dental health and indicators of oral hygiene among school-going children in a fluoride-endemic belt in the rural part of Chittoor district. The burden of dental caries in the study population was 36%, with a marginally higher proportion in females (38.5%), which is almost similar to a study conducted in Nalgonda district, a fluoride-endemic region among school children, which reported 43.4% dental caries with a higher prevalence in females [11]. However, it is a much lesser estimate than the national pooled estimates of 52% in the age group of 3-18 years [16], whereas another study from a fluoride-endemic zone of Andhra Pradesh reported a prevalence of 56.3% [17].

Almost 70% of the study children had good oral hygiene, and only 12% had gingivitis, indicating good oral hygiene practices. In the study, good oral hygiene and mild fluorosis were associated with protection against dental caries by 43% and 28%, respectively. Similar findings of a lower proportion of dental caries among people with mild fluorosis have been reported in different geographical regions, including Tamil Nadu, Gujarat, and Japan by Ushanthika et al. [18] and Nivetha et al. [19], Kotecha et al. [20], and Tsutsui et al. [21], respectively. These findings indicate that milder forms of dental fluorosis do not compromise oral health or function and may in turn enhance resistance to decay due to the increased fluoride content in enamel [22].

Although Chittoor district is endemic for fluorosis, the study documented 29% of fluorosis among the study children, which is comparable to the national pooled estimates of 34.5% as reported by a meta-analysis by Mittal et al. in 2025 [23], but far lesser than a study from Nalgonda, a fluoride-endemic belt, which documented 71.5% of dental fluorosis [17]. Mittal et al.'s [23] study showed a higher prevalence of fluorosis in males, whereas this study did not show any significant predominance in males. Only very mild form of fluorosis was identified among the study children. A study conducted in Chittoor district quantified fluoride in drinking water and demonstrated fluoride levels ranging from 0.4 to 2.8 mg/L [24], indicating different gradient of exposure in geographic locations.

Interestingly, the level of healthcare utilization was just 2.2%, which highlights the necessity to bring awareness of healthcare facilities and the importance of seeking treatment. Primary health care is the pivotal point of contact between healthcare professionals and patients. To achieve efficient primary care, coordinated health systems should be established, providing accessibility to a full range of affordable basic health services and to higher levels of treatment, if required, through referrals from primary care. The use of dental services in India is hugely influenced by social, economic, and individual factors. There is a skewed distribution of accessibility and availability of oral treatment, with a stark rural-urban divide in India. Financial constraints, lack of awareness (preventive and therapeutic), long distance to the nearest dental care facilities, lack of infrastructure, and lack of accountability are known factors for the impediment of healthcare utilization [25]. A meta-analysis reported that only 24% of Indian adults utilized dental care, with the highest estimate in south zone at 30% [26]. Therefore, even though the National Oral Health Initiative was started with a goal of delivering accessible, equitable, and cost-effective oral health care, especially in rural areas, the above estimates underscore the need for critically reviewing, identifying implementation gaps, and strengthening the existing oral health policies and strategies.

This study had few limitations. Due to time constraints in this school-based evaluations, a detailed socioeconomic and dietary evaluation could not be done. The study population included children who had various sources of drinking water with different levels of exposure to fluoride. This study could not measure the water fluoride levels in different sources, although a study conducted in Chittoor district demonstrated varied levels of fluoride content in different areas [24]. Hence, we could only extrapolate that the study children may be staying in villages with mild to moderately high levels of fluoride.

## Conclusions

The purpose of this study was to estimate the dental disease burden, oral hygiene practices, and health utilization among the school-going adolescents in a fluoride-endemic belt of rural Andhra Pradesh. It was encouraging to find that the overall burden of dental caries, gingivitis, and malocclusion was low, and oral hygiene was good in the study population. Good oral hygiene and mild non-pitted fluorosis were associated with protection against dental caries. One of the valuable insights from this study was low healthcare utilization among children in the region. Although identifying the attributing factors was beyond the scope of this study, there is a need to explore in the future. Promoting school-based oral health programmes play a pivotal role in spreading knowledge and awareness among students about oral health, diseases, and their consequences. In addition, health systems should support capacity building of primary care facilities to make dental treatment accessible, available, and affordable to improve the health-seeking behavior in the rural areas.
